# The role of feedback in the low-prevalence effect: a meta-analysis of criterion shifts in visual search

**DOI:** 10.1186/s41235-026-00747-5

**Published:** 2026-07-29

**Authors:** Vincent D. Caruso, Gavin Q. Fox, Nancy B. Carlisle, Patrick H. Cox

**Affiliations:** Department of Psychology, Lehigh University, Chandler-Ullmann Hall Room 120, 17 Memorial Drive East, Bethlehem, PA 18015 USA

**Keywords:** Applied visual search, Signal detection theory, Prevalence-induced concept change, Robust variance estimation

## Abstract

In high-stakes visual search tasks, such as reading a medical image or screening luggage at an airport security checkpoint, targets are often rare, leading to disproportionately high miss rates—a phenomenon known as the low-prevalence effect (LPE). While the LPE is traditionally attributed to conservative shifts in decision criterion (c), recent research on prevalence-induced concept change (PICC) suggests that low prevalence can lead to liberal decision criterion shifts in certain contexts. We propose that trial-level performance feedback is a critical mechanism governing these divergent outcomes. We conducted a meta-analysis of 62 effects from 29 publications, using robust variance estimation (RVE) to account for statistical dependencies. Our results confirm a large and reliable conservative criterion shift under low-prevalence visual search conditions, with no consistent effect on sensitivity (d’). Critically, meta-regression revealed that trial-level feedback significantly moderated the magnitude of these shifts; the presence of immediate feedback amplified the LPE, whereas its absence mitigated it. Additionally, the magnitude of the shift was tied to the numerical difference in prevalence, but remained robust across variations in stimulus type, observer expertise, and time constraints. These findings suggest that trial-level feedback reinforces expectations of target scarcity, amplifying the conservative biases that drive the LPE. This resolves theoretical discrepancies between the LPE in visual search and PICC findings in categorization and highlights a potential “intervention paradox”: While generally intended to improve accuracy, standard trial-level feedback may unintentionally exacerbate misses in rare target detection.

**Supplementary Information:**

The online version contains supplementary material available at https://doi.org/10.1186/s41235-026-00747-5.

## Significance statement

In many critical real-world visual search environments like medical imaging or airport security, the most important targets are often the rarest. Decades of research on the low-prevalence effect have shown that as targets become rare, observers develop a bias toward reporting them as absent, which leads to an increase in missed target detections. This meta-analysis shows that feedback about search accuracy, a tool often used to improve search performance, actually exacerbates the increase in missed targets in low-prevalence settings by strengthening this reporting bias. By consistently reinforcing the absence of a target in low-prevalence search, feedback appears to bolster the observer's expectation of target scarcity rather than sharpening their target detection when searching for these proverbial needles in a haystack. These meta-analytic results have implications for how professional visual searchers should be trained, as well as how basic science studies of low-prevalence visual search will generalize differently in applied settings depending on the availability and type of feedback.

**Supplementary Information:**

The online version contains supplementary material available at https://doi.org/10.1186/s41235-026-00747-5.

## Introduction

Visual search—the act of locating a target item among distractors—is a core component of human cognition, supporting a vast array of tasks in scenarios that range from low stakes (e.g., finding a pen on a cluttered desk) to high stakes (e.g., airport baggage screening and medical imaging). In applied contexts such as radiology and transportation security, high accuracy is imperative given the potentially life-threatening consequences of search errors. A defining feature in these environments is that targets are exceptionally rare, with prevalence rates well below 1% in domains like cancer screening (Fenton et al., 2007) and airport baggage checks (Transportation Security Administration, 2024). This target scarcity creates a striking disconnect between real-world search contexts and many laboratory-based tasks, which typically use a baseline target prevalence of 50%.

Questions surrounding the effects of target prevalence on diagnostic performance were first addressed in the medical image perception literature. Early retrospective and theoretical work suggested that low prevalence could systematically bias radiological decisions and complicate the interpretation of sensitivity and specificity measures (Kundel, 1982, 2000). Subsequent laboratory studies using radiologists and clinical imaging stimuli produced mixed findings regarding the extent to which prevalence altered target detectability versus decision thresholds (e.g., Ethel & Manning, 2001; Gur et al., 2003). Meanwhile, motivated by the prevalence question in airport baggage screening, cognitive psychology research carried out studies aimed at isolating the mechanisms underlying prevalence-related search errors. This experimental work characterized the substantial influence that target prevalence can exert on visual search performance. Specifically, in low-prevalence conditions, observers disproportionately fail to detect the few targets present, a phenomenon known as the low-prevalence effect (LPE; Wolfe et al., 2005). Across a wide range of tasks and stimuli, miss rates have been shown to rise drastically, from 10% to upwards of 30–40% through the manipulation of target prevalence alone (e.g., Peltier & Becker, 2016; Wolfe et al., 2007).

The LPE is effectively characterized using a signal detection theory framework (Green & Swets, 1966; Macmillan & Creelman, 2005). Signal detection theory separates performance in binary decision tasks into sensitivity (d’) and criterion (c). Sensitivity reflects the perceptual separability of targets from distractors.^1^ Criterion represents the observer’s decision threshold for determining “target-present.” Criterion is used to measure an observer’s decision bias, with any tendency toward either target-absent or target-present responses reflected in a more positive or negative criterion, respectively. These two measures, sensitivity and criterion, are theoretically independent constructs in signal detection theory.

A consistent finding throughout the LPE literature is that changes in performance are driven primarily by shifts in decision criterion, rather than a change in target detection sensitivity. The dual threshold model, a leading account of prevalence-related changes in visual search, outlines two separate decision processes: an identification threshold representing the amount of evidence required to classify an item as the target, which is of primary interest here, and a quitting threshold representing the amount of time an observer will conduct a search before responding target-absent for a given trial (Wolfe & Van Wert, 2010; Wolfe et al., 2007). As target prevalence decreases, observers adopt increasingly conservative criteria—a shift in the first threshold mentioned above—biasing responses toward “target-absent” decisions, which substantially increases miss rates, with false alarm rates typically decreasing concomitantly.

### Feedback

Explicit accuracy feedback plays a critical role in shaping observers’ responses in search tasks (Boot et al., 2009). Within the low-prevalence literature, feedback has been employed as a tool to mitigate or reshape the LPE through a range of experimental manipulations. J. Wolfe and colleagues demonstrated that inserting a brief block of high-prevalence search, accompanied by trial-level accuracy feedback, among longer blocks of low-prevalence search without feedback promoted a more liberal decision criterion that persisted into subsequent low-prevalence blocks (Wolfe et al., 2007, 2013). Schwark et al. (2012) demonstrated that false feedback emphasizing miss errors (i.e., reclassifying a subset of correct rejections as misses) can encourage observers to adopt a more liberal criterion and enhance target detection under low prevalence. The feedback manipulations in these studies operated by adjusting observers’ expectations of target scarcity, which has been shown to substantially influence search performance (Cox et al., 2021). Paradigms that selectively provided feedback only during high-prevalence bursts (Wolfe et al., 2007, 2013) encouraged observers to rely on these blocks to estimate the overall target prevalence, leading to an expectation of more frequent targets that persisted through subsequent low-prevalence blocks. Schwark and colleagues’ false feedback manipulation achieved a similar outcome more directly, by explicitly decoupling observers’ perceived prevalence from the true distribution of trials.

Despite the previous use of feedback-based manipulations, direct experimental comparisons of low-prevalence effects in visual search with versus without feedback are relatively scarce. For over a decade, the primary comparison came from a cross-study analysis by Van Wert, Wolfe, and colleagues (Van Wert et al., 2009; Wolfe et al., 2007). They explain that their 2007 and 2009 studies employed highly similar methodologies, with the most notable difference being the presence of trial-level feedback in the earlier study (Wolfe et al., 2007). They reported that the prevalence effect was more pronounced when feedback was provided, with observers adopting more conservative criteria and exhibiting higher miss rates (Van Wert et al., 2009; Wolfe et al., 2007).

On the other hand, the impacts of feedback on prevalence effects have been closely examined in search-adjacent tasks such as identification and classification. Levari et al. (2018) introduced the concept of prevalence-induced concept change (PICC), demonstrating that when observers categorize stimuli drawn from a continuous feature space one at a time, decreasing the prevalence of one of the categories in the absence of feedback leads to liberal shifts in that category’s inclusion criterion (in a sense, the opposite of the LPE). Lyu et al. (2021) extended these results, demonstrating the central role of feedback in such tasks. They again showed that withholding feedback reliably produced liberal shifts in a category’s inclusion criterion (i.e., PICC), whereas providing feedback produced conservative shifts (i.e., the LPE). Decreases in target prevalence in the absence of feedback and the resulting increasing proportion of target-absent responses may lead observers to doubt their internal representation of the target, resulting in the liberal shifts characteristic of PICC (Levari et al., 2018). When accuracy feedback is provided, however, the predominance of confirmed correct rejections biases observers toward an overly conservative criterion characteristic of the LPE (Lyu et al., 2021). Similarly, Wolfe (2022) found feedback to be the determining factor between observing the LPE and PICC in a low-prevalence skin lesion classification task. Although the PICC observed was weak in magnitude, response criteria were significantly more liberal in no-feedback conditions than when feedback was provided. Additionally, Papesh et al. (2018) observed the low-prevalence effect in an unfamiliar face-matching task only when accuracy feedback was provided. The authors attributed this pattern to feedback informing observers about the base rates of mismatching faces, consistent with feedback manipulations in visual search that shape expectations about target prevalence.

Recently, this feedback-driven modulation of prevalence-induced criterion shifts has been observed across three applied search tasks (Kunar et al., 2025; Lyu et al., 2025; Song & Wolfe, 2024). Song and Wolfe (2024) found that in a dynamic road hazard detection task, low-prevalence effects emerged only when feedback was provided; when feedback was withheld, observers exhibited comparatively liberal response biases, although these shifts were not large enough to constitute PICC. Lyu et al. (2025) reported the same interaction in a blast cell identification paradigm (Lyu et al., 2025). Across multiple experiments, Lyu and colleagues demonstrated that feedback produced robust conservative criterion shifts under low prevalence in both novices and experts, whereas low-prevalence search without feedback did not significantly shift observers’ decision criteria in either group. Notably, across these paradigms, unlike in categorization tasks (Levari et al., 2018; Lyu et al., 2021), withholding feedback eliminated conservative low-prevalence biases without producing a reliable liberal shift.

Kunar et al. (2025) further demonstrated that prevalence-related criterion shifts can be actively reversed through manipulated feedback. In a low-prevalence mammography screening task, exaggerated block-level feedback emphasizing miss errors led observers to adopt an overly liberal criterion, effectively reversing the typical LPE pattern. Extending the findings of Schwark et al. (2012), these results suggest that criterion shifts are driven not simply by the presence of feedback, but by feedback’s role in shaping observers’ expectations about target prevalence and their own distribution of misses and false alarms.

Together, the previous literature suggests that feedback, through its influence on observers’ expectations of target base rates, is an important factor in shaping low-prevalence effects (alongside a number of other factors like computer-aided detection (Kunar, 2022), reward (Hadjipanayi et al., 2023), AI cues (Kunar et al., 2024), and prepotent motor responses (Fleck & Mitroff, 2007). While interactions between PICC and the LPE have been explicitly examined in identification and classification tasks, only recently have paradigms directly examined feedback’s modulating role on prevalence effects in search paradigms. Across experiment comparisons of low-prevalence search with and without feedback in the visual search literature (e.g., Wolfe et al., 2007 vs. Van Wert et al., 2009), alongside demonstrations of PICC–LPE interchanges in search-adjacent perceptual decision-making tasks (as well as some applied visual search tasks such as Song and Wolfe (2024)), suggest that even if PICC does not reliably emerge in visual search, feedback should, at minimum, produce measurable increases in the magnitude of prevalence-related criterion shifts.

### Current study

Although the low-prevalence effect has been extensively documented, including a prior meta-analysis (Horowitz, 2017), no quantitative syntheses and limited primary research have directly examined whether and how feedback moderates prevalence-induced criterion shifts in visual search. Horowitz (2017) established the robustness of the LPE and confirmed that it is driven by shifts in the decision criterion rather than sensitivity; however, feedback was not evaluated as a potential moderator. The present meta-analysis builds upon this foundation by systematically testing feedback as a moderator across the broader visual search literature and by employing robust variance estimation (RVE) to appropriately weight statistically dependent effects within and across studies.

Our primary research question concerned the moderating effects of feedback on prevalence-induced criterion shifts. We hypothesized that trial-level feedback would amplify conservative criterion shifts under low prevalence relative to conditions without feedback. We also examined block feedback to distinguish immediate trial-by-trial feedback from delayed performance information. While block-level feedback summaries are less commonly employed and far less studied, their inclusion allows us to test whether the immediacy of feedback is critical for shaping prevalence-driven biases.

In addition to feedback, we investigated whether search systematically varied based on stimulus type, observer expertise, trial-level time constraints, and the numerical prevalence difference between conditions. Stimulus type and observer expertise were included to replicate and extend the findings of Horowitz’s (2017) meta-analysis by testing them on a larger and more diverse sample of studies. We included two additional regressions not included in the previous meta-analysis: trial-level time constraints and the numerical prevalence difference between conditions.

Consistent with prior meta-analytic findings (Horowitz, 2017), we hypothesized that simulated baggage would show more positive criterion effects (i.e., less of a LPE), while shape-based searches would show more negative ones (i.e., more of a LPE). These hypothesized differences are based on the fact that simpler stimuli might lead to more motor prepotency (Fleck & Mitroff, 2007; Rich et al., 2008) and simple stimuli might have more straightforward search templates, whereas more complex real-world stimuli might require searching for categories of things via hybrid search (Cunningham & Wolfe, 2014; Wolfe, 2012) Also in line with Horowitz (2017), we predicted that observer expertise would not reliably moderate either criterion or sensitivity. Prior work has demonstrated that professional observers, both radiologists and transportation security officers, exhibit similar prevalence-related search patterns as novices (Evans et al., 2011; Nakashima et al., 2015; Wolfe et al., 2013). Moving onto the novel meta-regressions, we predicted that larger numerical differences in prevalence between conditions would be associated with greater criterion shifts, in line with prior proposals (Horowitz, 2017; Kramer et al., 2022; Wolfe & Van Wert, 2010). Finally, for time constraints, we expected shorter trial durations to be associated with more conservative criterion shifts through acclimation to faster responses based on less evidence; prior work has shown that forcibly delaying responses can mitigate criterion shifts, so requiring faster responses may elicit the opposite effect (Rich et al., 2008). While our central focus was on criterion shifts, we also examined sensitivity through both summary effects and within-moderator analyses. In line with Horowitz’s (2017) findings, we expected no systematic differences in sensitivity as a function of stimulus type or observer expertise. We also expected no significant variation based on the numerical prevalence difference. However, we hypothesized that tighter time constraints would be associated with more negative sensitivity effects.

## Methods

### Eligibility criteria

**Fig. 1:**
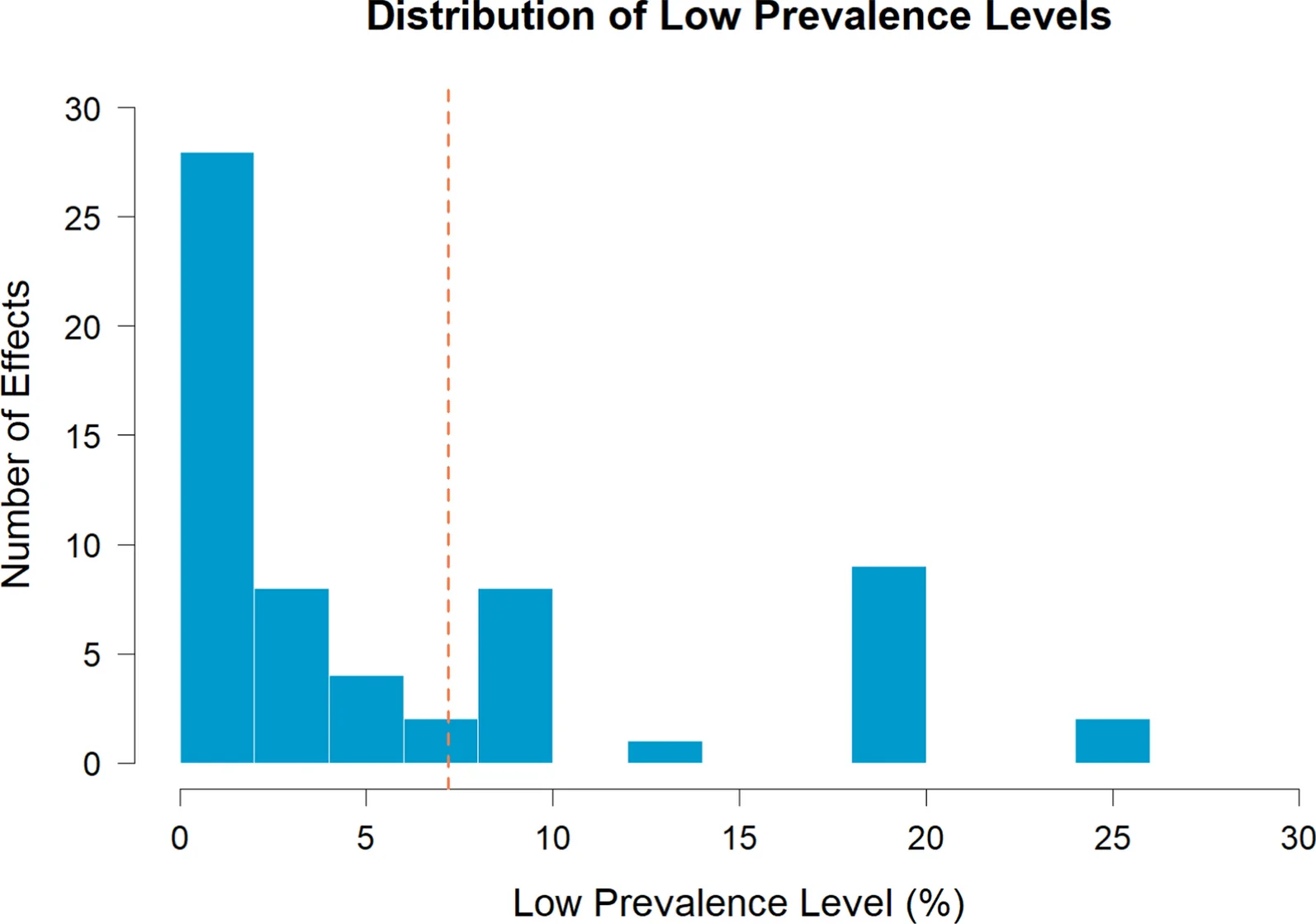
Distribution of included effects across the low-prevalence range. Mean low prevalence level is indicated by the dashed vertical line

- *Study designs* Empirical studies utilizing either cross-sectional or longitudinal designs were included.
- *Language and publication status* Only peer-reviewed, published studies written in English were eligible. No restrictions were placed on the year of publication or country of origin.
- *Task characteristics* Search tasks were required to feature both target-present and target-absent trials. Studies had to include a low-prevalence condition (1–30%; Fig. 1) and a medium-prevalence condition (40–60%). We chose these ranges because target prevalence of around 50% is widely used as the experimental baseline in the prevalence literature, lower prevalence levels are most often examined due to their implications in applied settings, while higher prevalence levels are examined less frequently. Designs featuring multiple targets of varying relative prevalence were included only when separate response keys were used for each target.
- *Signal detection* Studies were required to report the signal detection parameters, sensitivity (d’) and criterion (c), or provide data through which they could be derived. When such data were not available, we contacted the corresponding authors to request it.
- *Study size* There were no restrictions on sample size or number of blocks and trials per condition.

### Selection and search strategy

A systematic literature search was conducted using Google Scholar and PubMed to identify studies meeting the eligibility criteria outlined above. The first round of search, citation tracking, was conducted using Google Scholar starting in January 2025, focusing on two influential publications in the study of prevalence effects in visual search. The first, Wolfe et al. (2005), “Rare items often missed in visual searches,” is widely regarded as foundational to the study of low-prevalence changes in visual search; a total of 782 citing articles were screened. The second, Horowitz (2017), “Prevalence in visual search: From the clinic to the lab and back again,” is a prior review and meta-analysis of low-prevalence search; 79 citing articles were screened in addition to the 13 studies originally included in that analysis. Following citation tracking, a keyword-based search using the Boolean string (“visual search” OR “target detection”) AND (prevalen* OR target frequen* OR rare targe*) was conducted on PubMed starting in July 2025. Both searches, Google Scholar and PubMed, concluded in December 2025.

**Fig. 2:**
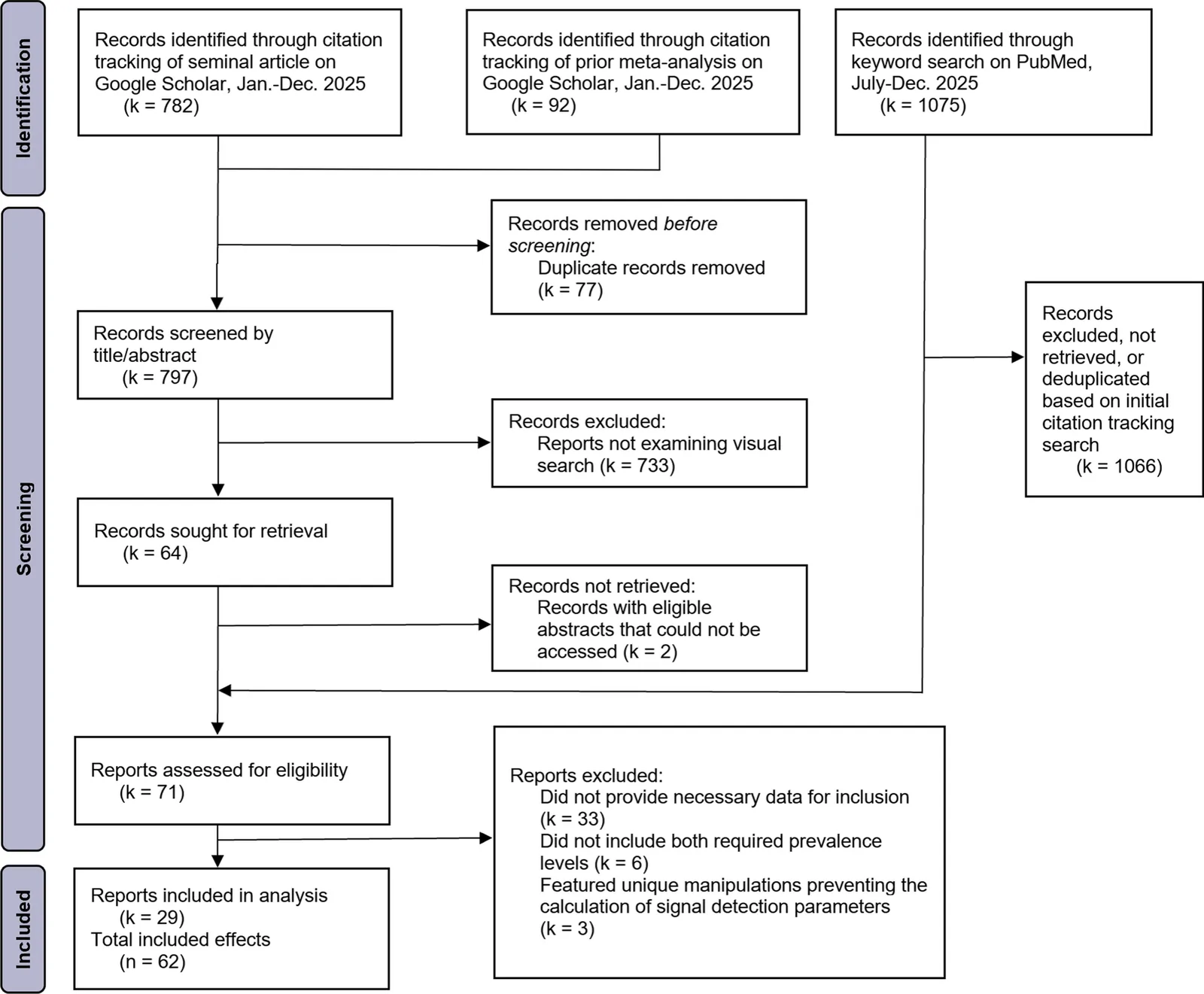
PRISMA flow diagram showing the study screening process

Title and abstract screening were performed manually. Articles were flagged for full-text review if the title or abstract contained relevant terms such as “search,” “prevalence,” “target,” “rare,” or “detection.” Methods sections of flagged articles were examined to determine whether they met the eligibility criteria outlined above. Citation tracking yielded the majority of included studies. A total of 1948 articles were screened before deduplication. Screening was conducted by a single reviewer following the prespecified inclusion criteria. In cases where inclusion was unclear, the study was discussed with two additional reviewers until a consensus was reached. The full screening process is depicted in the PRISMA 2020 flow diagram (Fig. 2).

### Data collection

For all eligible studies, a comprehensive set of data was extracted outlining study identification, sample characteristics, experimental design, and visual search task properties. Study identification information includes the study name, authors, publication year, and citation. Sample characteristics include the number of participants and their level of search expertise. Study design information consisted of the experimental design (i.e., within- or between-subject designs) and whether the experiment featured multiple experimental conditions beyond the manipulation of prevalence. Finally, detailed characteristics of the visual search task were collected, including feedback, stimulus type, the number of blocks and trials per block, the target prevalence of each block, and time constraints on trial duration.

The two outcomes of interest were the signal detection parameters sensitivity (d’) and criterion (c). Five search characteristics (feedback, stimulus type, observer expertise, time constraints, and numerical prevalence difference between conditions) were examined as potential moderators of prevalence-related patterns in visual search performance. For categorical moderators, a reference category was selected to serve as a baseline for comparison. Feedback, defined as performance-related information provided to the observer, was coded as no feedback (reference group), block-level feedback, or trial-level feedback. Stimulus type was categorized as medical images, simulated baggage, objects, shapes, or letters, and observer expertise was categorized as novice or professional (i.e., radiologists or transportation security officers). Consistent with Horowitz (2017), medical images and novice observers were used as the reference groups for stimulus type and observer expertise, respectively (Horowitz, 2017). Time constraints were split into no time limit (reference group), standard time limits (8–12 s), and short time limits (less than 6 s). Prevalence difference was treated as a continuous variable representing the numerical difference in target prevalence between compared conditions, ranging from 25 to 48%.

### Analysis plan

All studies included in the final synthesis—62 effects (i.e., medium–low comparisons) for each signal detection parameter, across 29 publications (Table 1)—directly reported or provided sufficient data to compute sensitivity (d’) and criterion (c) under both required prevalence conditions. As a result, all eligible studies contributed to the overall meta-analyses of both outcome measures.

**Table 1:** Publications included in the present meta-analysis, previous meta-analysis, or both

Present analysis	Both	Horowitz (2017)
Beanland et al. (2016)	Evans et al. (2011)	Egglin and Feinstein (1996)
Buser et al. (2020)	Wolfe et al. (2007)	Ishibashi et al. (2011)
Corbett and Munneke (2018)	Nakashima et al. (2013)	Kunar et al. (2010)
Drew and Williams (2017)	Nakashima et al. (2015)	Menneer et al. (2010)
Fleck and Mitroff (2007)	Rich et al. (2008)	Reed et al. (2014)
Gillies and Kosovicheva (2025)	Russel and Kunar (2012)	Tollner et al. (2012)
Goodhew and Edwards (2024A, 2024B)		Yan et al. (2010)
Goodhew and Edwards (2024A, 2024B)		
Hebert et al. (2020)		
Ishibashi and Kita (2014)		
Kang et al. (2024)		
Kunar et al. (2017)		
Kunar et al. (2021)		
Lyu et al. (2025)		
Madhavan et al. (2013)		
Miyazaki (2015)		
Moher (2019)		
Pinto and Papesh (2024)		
Schwark et al. (2012)		
Thomson and Goodhew (2021)		
Van Wert et al. (2009)		
Zsido et al. (2024)		

Before synthesis, all data outcomes were converted to Hedges’ *g* (also known as D unbiased) using the standard formula for paired or independent designs, as appropriate (Cumming, 2012). When studies included multiple conditions or unique trial types upon which data could be meaningfully split for separate prevalence comparisons, effect sizes were calculated for each condition separately to parallel prior meta-analyses (Horowitz, 2017). These cases were handled using robust variance estimation (RVE) to account for the dependency between effects within studies (Tanner-Smith et al., 2016).

When converting results to Hedges’ *g*, we treated medium prevalence as the treatment group and low prevalence as the control. Our final effects represented the difference scores of medium minus low prevalence for each signal detection parameter. An effect near zero suggests that, on average, the given parameter did not vary between prevalence conditions. A positive effect indicates the parameter is greater under medium prevalence, while a negative value indicates it is greater under low prevalence.

When studies provided group means, standard deviations, and sample sizes, Hedges’ *g* was calculated directly. When only test statistics (*t* or *F*) were reported, we used standard conversion formulas. For each of these cases, effect sizes were calculated using the Campbell Collaboration Practical Meta-Analysis Effect Size Calculator (Wilson, 2023). For within-subjects designs where the correlation (*r*) between conditions was unknown, we conservatively calculated Hedges’ *g* assuming independent samples (which underestimated the true effect size), consistent with previous meta-analyses (Horowitz, 2017).

For studies providing raw accuracy data, hit and false alarm rates were calculated for each participant under each prevalence level; these rates were used to calculate the signal detection parameters and ultimately Hedges’ *g* for each parameter. In some cases, observers demonstrated perfect hit or false alarm rates that would have resulted in extreme values for the signal detection parameters due to the use of z-scores. In these cases, we used the “1/(2*N*) rule,” which converts proportions of 0 and 1 into 1/(2*N*) and 1–1/(2*N*), respectively, with *N* being the number of target-present or target-absent trials on which the proportion is based (Macmillan & Creelman, 2005; Hautus, 1995).

### Tabulation and graphics

All visualizations and statistical analyses were conducted in R (v4.3.0) alongside several imported packages. *dplyr* was used to calculate hit and false alarm rates from raw accuracy data frames, and *esci* was used to compute Hedges’ *g* from participant-level d′ and c values. Visualizations were created using *metafor* for funnel plots, *vioplot* for violin plots, and *ggplot2* for histograms and scatter plots.

Effect sizes and net effects were summarized using forest plots generated via the *robumeta* package (Fisher & Tipton, 2015). Each forest plot grouped effects by study and displayed the Hedges’ *g* estimate alongside its 95% confidence interval and weight. When multiple effect sizes were drawn from the same publication, individual effects were labeled using either a condition identifier or the experiment number from the original study. Before synthesis, violin plots were screened for potential outliers in effect sizes for each parameter, defined as values exceeding 1.5 times the interquartile range beyond the first or third quartile; no such outliers were detected.

Robust variance estimation (RVE) was performed using *robumeta*. All syntheses were conducted using RVE to handle cases in which a single publication featured multiple studies from which effects could be drawn. RVE can accommodate these hierarchically dependent effect sizes by estimating robust standard errors that account for within-study correlations (Tanner-Smith et al., 2016). We used random-effects models using hierarchical weights and a default *ρ* = 0.8 after running sensitivity analyses that determined the choice of rho (*ρ*) had no impact on the results. These sensitivity analyses were conducted to determine whether the between-study variance (*τ*^2^) and the average effect size in each primary analysis differed as a function of *ρ*, or how much variance was shared among effects within the same study (e.g., 0 = completely independent; 1 = completely dependent). Given that rho is an estimate of the relationships among effect sizes within a study and influences standard errors, it is important to ensure that our results are significant regardless of how closely effects within a study are related. Confidence intervals were calculated using the small sample correction recommended by Tipton (2015).

An estimate of between-study heterogeneity was obtained from intercept-only random-effects models. Between-study variance (*τ*^2^) was used to quantify the absolute magnitude of heterogeneity across studies, while *I*^*2*^ describes the proportion of total variability in effect sizes attributed to heterogeneity rather than sampling error (Borenstein et al., 2021; Higgins & Thompson, 2002). Prediction intervals were calculated for sensitivity and criterion to estimate both expected ranges of possible effects in future studies (Partlett & Riley, 2016; Deeks et al., 2024; Riley et al., 2011). Prediction intervals differ from confidence intervals in that they incorporate between-study variance to describe uncertainty around the distribution of future effects, while confidence intervals describe uncertainty surrounding the mean effect (Higgins et al., 2009). Because the effects are calculated as difference scores, a prediction interval centered near zero suggests that future studies may find a given parameter to be higher under either medium or low prevalence. A predominantly positive prediction interval indicates that future studies should expect to find the parameter is greater under medium prevalence, while a predominantly negative prediction interval suggests that it would be greater under low prevalence.

### Meta-regression models

Meta-regression models evaluated whether study-level characteristics moderated the relationship between prevalence and the signal detection parameters. Several studies in this meta-analysis did not include data for some moderators. Given that RVE requires complete data for all included moderators and is sensitive to missingness, we conducted meta-regression analyses with one moderator entered at a time and excluded studies missing the relevant data for each analysis. Moderation was considered significant when the *t*-test for the moderator was significant (*p* <.05) and d*f* > 4.

In the case of numerical prevalence difference as a continuous moderator, all studies were included. Meta-regressions for categorical variables were conducted to compare each non-reference category against the given variable’s corresponding reference group using the subset of studies where the relevant information was available. For example, in the regressions examining stimulus type, independent regressions were conducted to compare each stimulus type (simulated baggage, objects, shapes, and letters) against the reference group (medical images), resulting in four regressions for d’ and four regressions for c. The final set of meta-regressions consisted of 24 parallel models: 12 examining criterion and 12 sensitivity. Breaking these down by moderator: 8 examined stimulus type, 4 on observer expertise, 6 on feedback, 4 on time constraints, and 2 on the numerical prevalence difference.

### Assessment of publication bias

To assess small-study effects and publication bias, funnel plots were visually inspected for asymmetry for each signal detection parameter. Funnel plots display individual effect sizes plotted against their standard errors, with asymmetry potentially indicating selective reporting of larger or more significant effects (Field & Gillett, 2010; Light & Pillemar, 1986; Macaskill et al., 2001; Sterne et al., 2011). In addition, a robust extension of Egger’s regression test was conducted using RVE regression with standard error as a moderator, to statistically assess funnel plot asymmetry (Egger et al., 1997). As Egger’s test can be sensitive to between-study heterogeneity and dependent effect sizes, results are to be interpreted cautiously and used as a diagnostic rather than definitive test of bias.

## Results

### Primary meta-analyses

The summary of net criterion and sensitivity effects was based on 62 effect sizes for each parameter, spanning 29 publications. Our sample comprised a diverse range of studies employing various methodologies. Visual search tasks ranged from traditional T-among-Ls letter searches to more applied paradigms emulating real-world contexts, such as analyzing medical images or transportation security screening; a detailed account of all included studies can be found in the supplemental materials, Table S2. All aggregated data, analysis code, results, and figures are available on the project’s OSF page https://osf.io/rwx79/, with replication steps provided in the prevalence_meta_analysis directory.

**Fig. 3:**
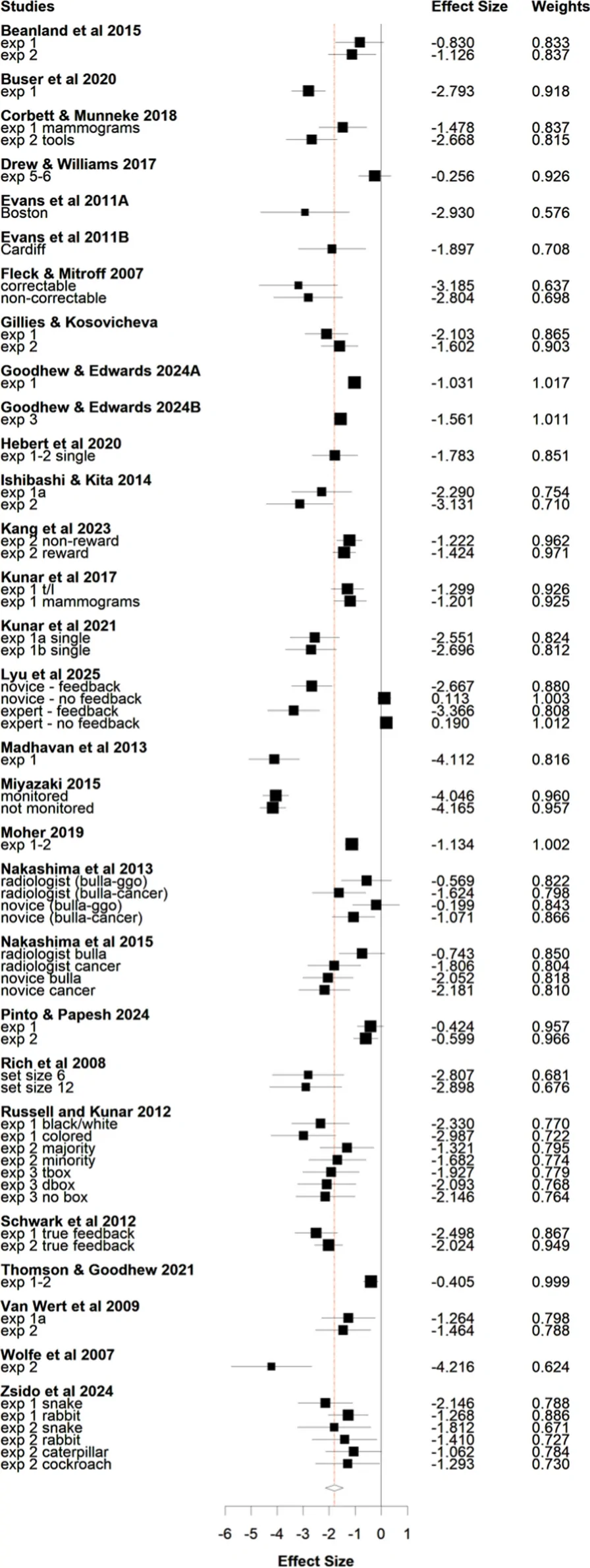
Forest plot of criterion effects using robust variance estimation. Effects are grouped by study and ordered alphabetically by first authors’ last names. Error bars represent 95% confidence intervals. Weights are based on effect variance and corrected for dependencies. Weights are depicted by the index size, and exact values are listed in the rightmost column. The summary effect is indicated by the dashed orange vertical line, and zero (no effect) is shown by the solid black line

There was a statistically significant overall effect for criterion (*g* = − 1.80, 95% CI [− 2.142, − 1.457], *p* <.0001, Fig. 3), indicating that observers adopted significantly more conservative response bias (i.e., favoring target-absent responses) under low-prevalence conditions. The 95% prediction interval for criterion effects ranges from − 3.498 to − 0.102, indicating that negative criterion effects are expected across future studies despite variability in task design and samples. Between-study heterogeneity for criterion was substantial (*τ*^2^ = 0.447; *I*^2^ = 63.72%), indicating meaningful variability across studies.

**Fig. 4:**
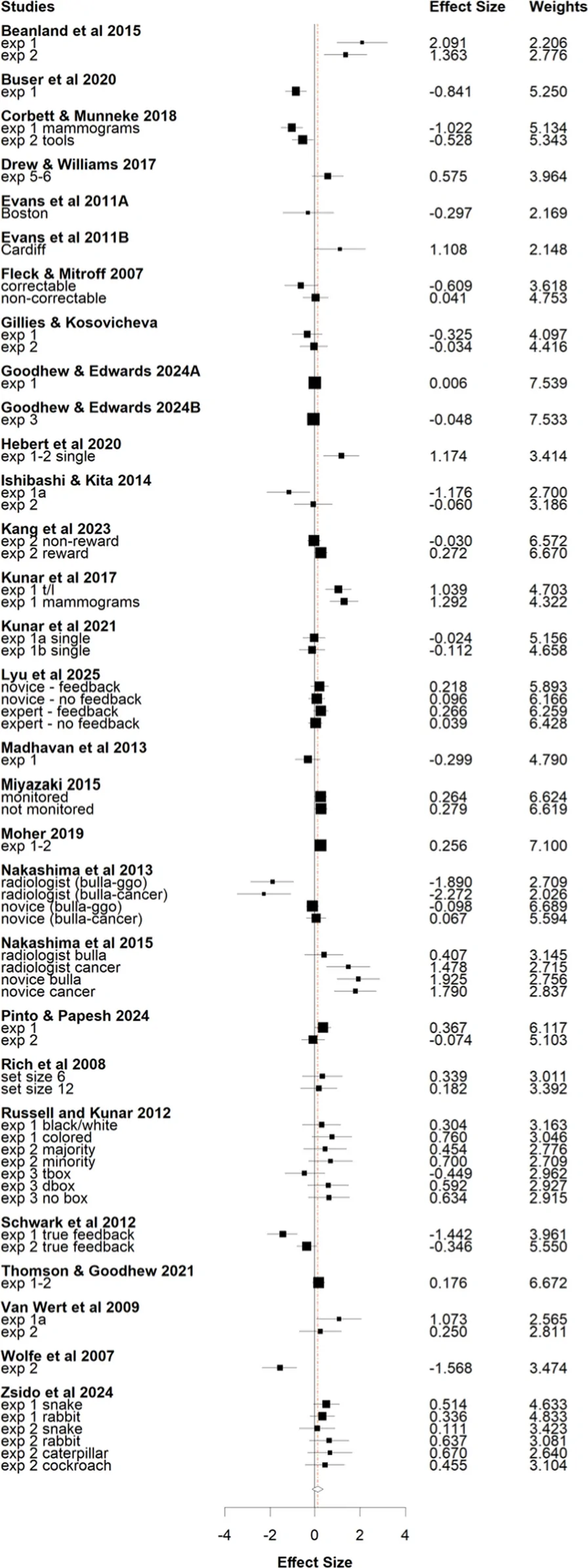
Forest plot of sensitivity effects using robust variance estimation. Effects are grouped by study and ordered alphabetically by first authors’ last names. Error bars represent 95% confidence intervals. Weights are based on effect variance and corrected for dependencies. Weights are depicted by the index size, and exact values are listed in the rightmost column. The summary effect is indicated by the dashed orange vertical line, and zero (no effect) is indicated by the solid black line

The net effect for sensitivity was not significant (*g* = 0.131, 95% CI [− 0.108, 0.371], *p* = 0.265, Fig. 4) with a prediction interval ranging from − 0.659 to 0.922. The wide prediction interval suggests that sensitivity differences may appear in individual studies but are more likely attributable to other task characteristics rather than prevalence alone. Between-study heterogeneity was moderate (*τ*^2^ = 0.150, *I*^2^ = 50.75%).

### Moderator analyses

**Fig. 5:**
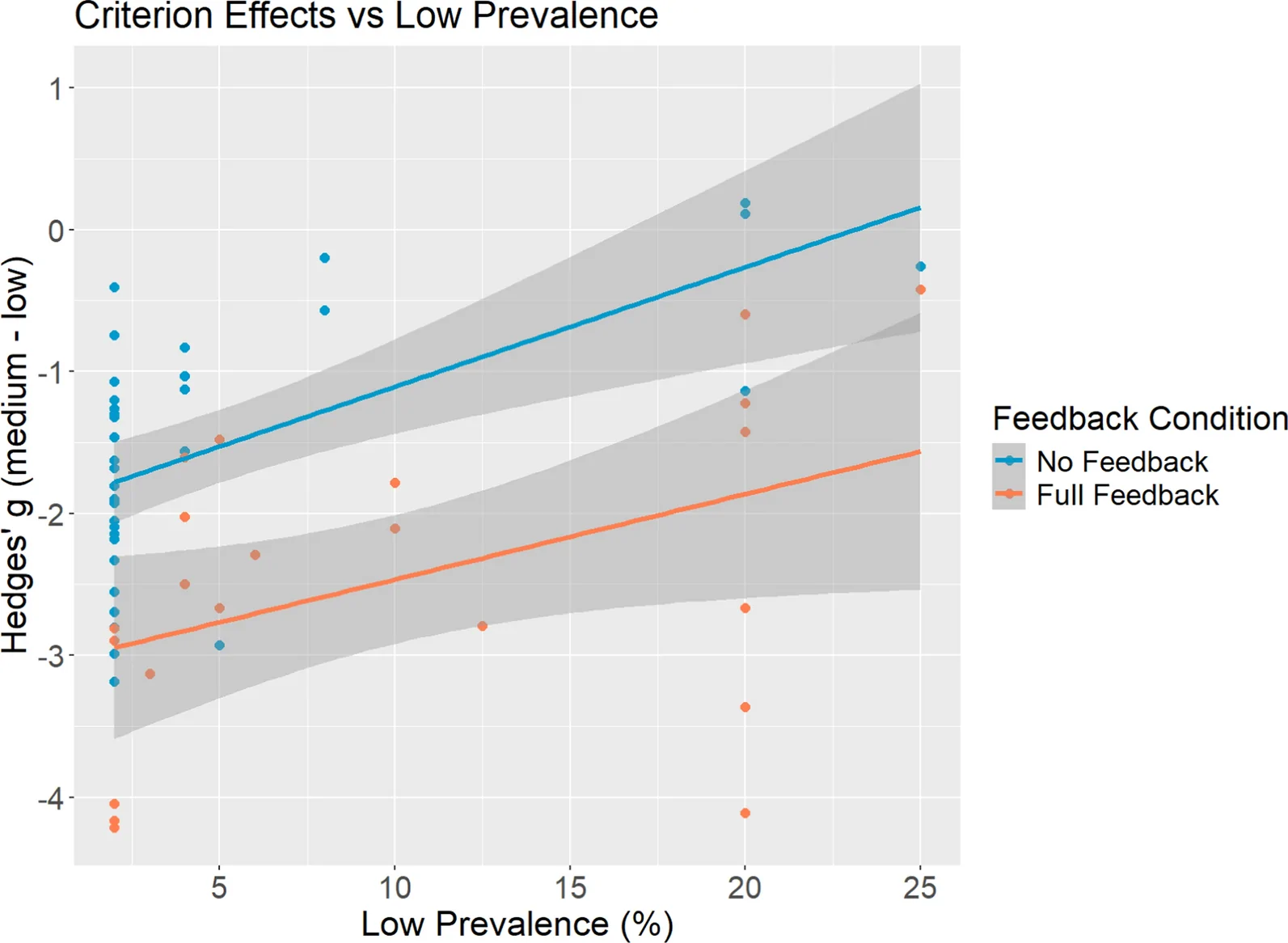
Scatter plot of criterion effects versus the low prevalence percentage. Trend lines are shown for trial-level feedback and no feedback separately, with 95% confidence intervals

All meta-regression results are reported in the supplemental materials, Table S1. Across the 24 meta-regression models, two significant effects emerged. First, the model examining the influence of trial-level feedback on criterion included *k* = 28 studies contributing *m* = 56 effects. Trial-level feedback was associated with larger criterion effects compared to no feedback (*b* = − 0.969, 95% CI [− 1.86, − 0.081], *p* =.034). The regression examining block feedback revealed no reliable effect on criterion (*b* = 0.140, 95% CI [− 0.266, 0.546], *p* =.454) based on *k* = 17 studies providing *m* = 40 effects. The second significant moderation was the numerical prevalence difference between conditions predicting criterion effects based on all *m* = 62 effects from the *k* = 29 studies (*b* = –0.054, 95% CI [− 0.096, − 0.013], *p* =.015) such that larger gaps in prevalence between conditions were associated with greater criterion shifts. Figure 5 shows a scatter plot illustrating the main effects of feedback and prevalence difference on the size of the criterion shift. A post hoc analysis testing whether the effect of the numerical low prevalence level on criterion was moderated by feedback condition (no feedback vs. full feedback) was not significant (*b* = − 0.010, 95% CI [− 0.136, 0.116], *p* =.855).

### Publication bias

**Fig. 6:**
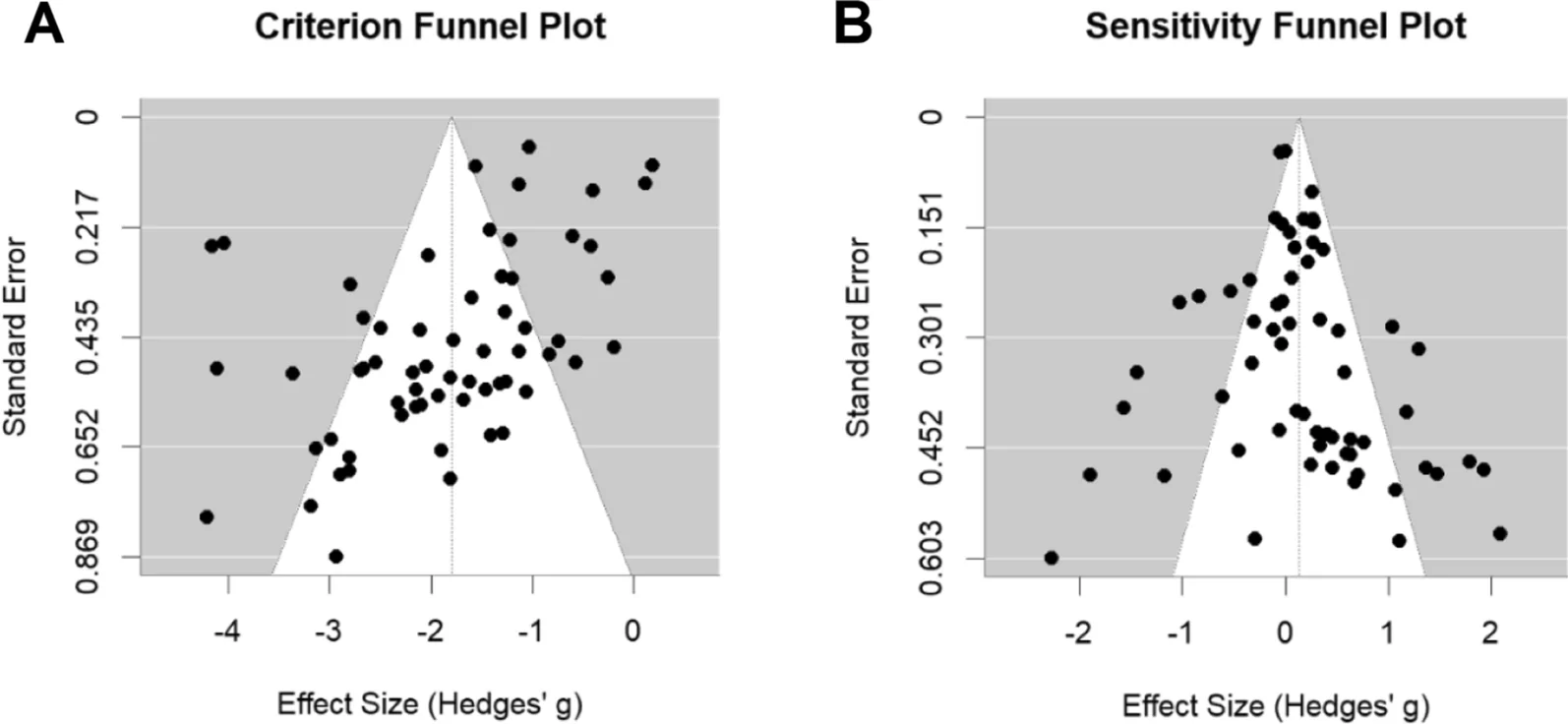
Funnel plots for criterion (**A**) and sensitivity (**B**) effects. Plots display effect size as a function of effect standard error. Larger, more precise studies are expected to cluster near the pooled effect, whereas smaller studies may show greater dispersion

After identifying visual asymmetry in the funnel plot for criterion (Fig. 6A), a robust RVE equivalent of Egger’s regression test was employed to quantify the risk of publication bias (Egger et al., 1997). For criterion, Egger’s test indicated a significant relationship between effect size and standard error (*β* = − 2.61, SE = 0.93, *t*(14.6) = − 2.82, *p* =.013). The significant negative slope suggests that studies with larger standard errors (e.g., smaller) tended to report more extreme effects relative to larger ones, consistent with potential small-study effects or publication bias. For sensitivity, Egger’s regression was not significant (*β* = 0.79, SE = 0.87, *t*(14.7) = 0.90, *p* =.382; Fig. 6B), providing no evidence of detectable funnel plot asymmetry. Between-study heterogeneity was substantial for criterion (*τ*^2^ = 0.633, *I*^2^ = 72.35%) and moderate for sensitivity (*τ*^2^ = 0.130, *I*^2^ = 48.84%).

## Discussion

Low-prevalence searches are common in high-stakes domains such as airport security screening, radiology, and military surveillance, where missed detections can have serious consequences. Prior work has consistently shown that the low-prevalence effect (LPE) in visual search primarily reflects a shift in decision criterion rather than changes in sensitivity (Wolfe et al., 2005, 2007; Horowitz, 2017), in line with early work showing signal probability affects only criterion during vigilance tasks where participants respond when a deviant auditory or visual stimulus is detected on a trial (Baddeley & Colquhoun, 1969; Colquhoun & Baddeley, 1967). The present meta-analysis provides robust quantitative confirmation of this pattern, demonstrating a large and reliable conservative criterion shift in low-prevalence conditions, alongside no consistent effect of prevalence on sensitivity. Furthermore, the prediction interval for criterion was fully negative [− 3.498, − 0.102], indicating that conservative shifts are expected across future low-prevalence search studies regardless of methodological distinctions and varying task characteristics.

Critically, this meta-analysis extends prior work by identifying accuracy feedback as a meaningful moderator of criterion shifts. Across studies, full trial-by-trial feedback was associated with significantly larger criterion effects compared to searches performed without feedback. This finding supports the hypothesis that trial-level feedback amplifies conservative response bias by reinforcing observers’ expectations about target scarcity. Under low-prevalence conditions, the most frequent feedback outcome is confirmation of correct rejections, which may disproportionately strengthen a bias toward target-absent responses and further elevate decision thresholds for reporting target presence. In contrast, in low-prevalence conditions without feedback, observers rely on their own response distributions, potentially interpreting frequent target-absent responses as uncertainty in their detection ability. Consequently, laboratory studies providing continuous trial-level feedback may overestimate the magnitude of the LPE relative to many applied search environments where immediate performance feedback is limited or absent. More broadly, these findings highlight the importance of matching key task characteristics, such as feedback structure, between laboratory paradigms and the real-world search environments they are intended to model.

The feedback effects described here align with prevalence-induced concept change (PICC) observed in categorization tasks (Levari et al., 2018). With respect to PICC, feedback has been shown to prevent liberal criterion shifts that emerge when observers perform categorization decisions in the absence of external performance information (Lyu et al., 2021). Relatedly, feedback has been shown to be necessary in eliciting low-prevalence effects in a face identity matching task (Papesh et al., 2018). In low-prevalence visual search, when feedback is provided, a stronger LPE is observed—likely because it eliminates ambiguity about the rarity of target-present trials. Recent work bridging PICC into applied visual search settings supports this interpretation, demonstrating that feedback reliably amplifies conservative criterion shifts in discriminating cancer cells from healthy white blood cells (Lyu et al., 2025) and hazard detection in driving (Song & Wolfe, 2024). Together, these findings suggest that feedback operates as a key mechanism linking prevalence, expectation, and response bias that extends through identification, discrimination, and classification into search paradigms.

The moderating effects of feedback on criterion, notably, were not seen in the regression examining block feedback. This distinction suggests that the influence of feedback on low-prevalence decision criteria may depend on the immediacy of the feedback rather than the mere provision of performance information. Trial-level feedback may more drastically shape observers’ expectations by repeatedly reinforcing the distribution of target-present and target-absent trials, whereas block-level summaries may lack the frequency or granularity to drive persistent criterion adjustment. There is also prior work that demonstrates that delayed feedback systematically disrupts implicit learning while leaving explicit learning intact (Smith et al., 2013). This points to the interesting implication that the LPE comes about as a result of implicit learning rather than an explicit strategy. It is important to note, however, that only six estimates could be included for block-level feedback, all originating from the same publication (Zsido et al., 2024). Accordingly, these findings should be interpreted with caution and highlight the need for more targeted interventions examining the influence of block-level feedback on the LPE.

The other significant result that emerged from the meta-regressions was that the numerical prevalence difference was associated with criterion shifts, such that larger gaps in prevalence between conditions were associated with greater shifts. This finding aligns with prior research, which has suggested that observers’ decision criterion is monotonically related to the exact prevalence level, rather than reflecting a general “low-prevalence” bias (Kramer et al., 2022; Mitroff & Biggs, 2013; Wolfe & Van Wert, 2010). Importantly, the relationship between prevalence difference and criterion shift appeared approximately linear across the 62 outcomes included in the analysis, and the effect did not differ as a function of feedback condition. This means that while feedback amplified the overall magnitude of the criterion shift, it did not alter the functional relationship between prevalence and response bias. Observing this pattern across a diverse set of task designs and methodological manipulations underscores the robustness of prevalence-driven criterion shifts.

It is important to note that the distribution of prevalence levels represented in the literature was highly uneven. Although we operationalized low prevalence broadly (1–30%) in line with prior work (Horowitz, 2017), the majority of included conditions fell between 2 and 5% (Fig. 1). Relatively few studies of low prevalence examined prevalence levels above 10% or below 1%; however, see Mitroff & Biggs (2013) for a study using extremely low prevalence rates matching those from critical real-world searches. This skew indicates that much of the empirical evidence for the linear low-prevalence effects we find here is concentrated in the very low range, leaving higher portions of the low-prevalence spectrum comparatively under-characterized. Given that prior work suggests criterion shifts vary continuously with the exact prevalence level (Kramer et al., 2022; Wolfe & Van Wert, 2010), future research would benefit from more systematic sampling across the full prevalence continuum, particularly in the sparsely studied upper low range where only subtle conservative bias would be expected. Such work would help determine whether the observed linear relationship extends uniformly across the full low-prevalence range, or whether understudied regimes could reveal nuances to the relationship between prevalence and criterion shifts (Kramer et al., 2022).

The regressions on stimulus type, observer expertise, and time constraints did not reveal significant moderation, suggesting that criterion shifts are highly robust across different task designs. The null results for stimulus type and observer expertise differ from those of Horowitz (2017), who reported that experiments using simulated baggage had more positive criterion effects, whereas shape-based searches produced more negative effects. This discrepancy likely reflects methodological differences. The current analysis included 62 effects from 29 publications and employed robust variance estimation (RVE) to adjust for statistical dependencies among multiple effects drawn from the same publication. By contrast, the earlier meta-analysis included 37 effects from 13 publications and did not describe procedures for handling such dependencies, raising the possibility that moderator estimates were influenced by correlated outcomes. Differences in the sets of included studies may also contribute to the divergent findings, as we were unable to obtain data from all studies included in Horowitz’s meta-analysis (Horowitz, 2017). A table showing the overlap between included publications is shown previously in Table 1. The null results of the regressions on time constraints may reflect the fact that tasks featuring strict response deadlines often differ systematically from unconstrained tasks in additional ways, such as smaller set sizes, more sparse arrays, higher target–distractor contrast, or other factors that are inherently more compatible with shorter trial durations.

### Limitations

With our novel meta-regression models, we aimed to account for nuances in task characteristics that explain variability in criterion shifts and outlier reports of sensitivity changes. Despite the current meta-analysis representing the largest synthesis of varying-prevalence visual search studies to date, the certainty of some moderator analyses is limited. Following robust variance estimation (RVE) guidelines (Fisher & Tipton, 2015), models with fewer than four degrees of freedom should not be considered reliable. While most of our analyses were sufficiently powered, the regression models examining the shapes category within stimulus type failed to meet the designated threshold, meaning we cannot meaningfully compare its average effects to the reference category of medical images. Future research should focus on determining whether these stimuli show systematically different prevalence effects.

Another limitation was the substantial number of eligible effects that could not be included due to insufficient data reporting. Of the roughly 140 effects identified that met the inclusion criteria, fewer than half could be analyzed. This loss of data reduced statistical power, limited the detection of significant moderators, and narrowed the precision of net effect estimates and prediction intervals. Improving the reporting and accessibility of trial-level accuracy data would enable future meta-analyses to include a broader portion of the literature, strengthening generalizability and interpretability (Godwin et al., 2025; National Academies of Sciences, Engineering, and Medicine, 2019).

Egger’s regression test indicated significant funnel plot asymmetry for the criterion, suggesting that the distribution of observed effects deviates from what would be expected under a model driven solely by sampling variability (Egger et al., 1997). In principle, such asymmetry may reflect publication bias, leading to an overestimation of the true effect. In the current analysis, larger and more precise studies exhibited the smallest criterion shifts, a pattern consistent with small-study effects. However, criterion effects showed substantial heterogeneity, indicating that the observed asymmetry may arise from genuine between-study differences in the absence of selective publication (Peters et al., 2010). Additionally, considering both the magnitude and significance of the net criterion effect, and the prediction interval spanning only negative values for criterion, even if some null or positive results went unpublished, it is unlikely that these results would overturn the observed negative criterion effect (Lipsey & Wilson, 2001). Taken together, these considerations suggest that the significant Egger’s test for criterion likely reflects heterogeneity across study designs and task manipulations rather than selective suppression of null or smaller effects.

### Conclusions

In conclusion, this meta-analysis provides strong quantitative support for the view that low target prevalence reliably produces conservative shifts in decision criterion without consistent changes in sensitivity. Beyond replicating this core pattern, the present findings identify accuracy feedback as a key moderator of criterion shifts, demonstrating that feedback systematically amplifies conservative response bias under low-prevalence conditions. These findings suggest that feedback is not merely a procedural detail, but a key theoretical component shaping prevalence-induced decision behavior. This points to the importance of considering the real-world low-prevalence search scenario you are attempting to model when deciding what type feedback, if any, to include in your design. Additionally, the magnitude of criterion shifts scales with the numerical difference in prevalence between conditions, supporting the perspective that prevalence effects do not represent a general “low-prevalence” bias, but rather a graded shift relative to the magnitude of the prevalence change. Together, these findings indicate feedback and the structure of prevalence manipulations jointly shape decision thresholds in visual search, and suggest both factors should be considered when translating laboratory findings to real-world visual search tasks.

## Supplementary Information

Supplemental Table 1.

 Supplemental Table 2.

## Data Availability

All data analyzed in this study were extracted from previously published articles cited in the References section. The aggregated dataset and analysis code supporting the findings of this study are publicly available on the project’s OSF repository: https://osf.io/rwx79/.

## Abbreviations

LPE: Low-prevalence effect
PICC: Prevalence-induced concept change
RVE: Robust variance estimation

## Footnotes

1 Importantly, the signal detection definition of sensitivity is distinct from its usage in medical literature, where it signifies the true positive rate (i.e., hit rate).

## Declarations

### Open practices statement

All aggregated data and analysis scripts supporting the findings of this study are publicly available on the project’s OSF page https://osf.io/rwx79/. The study protocol and analysis plan were not preregistered.
