# Supplementary material for: The role of feedback in the low-prevalence effect: a meta-analysis of criterion shifts in visual search
Source: Cogn Res Princ Implic. 2026 Jul 29;11:55. doi: 10.1186/s41235-026-00747-5 (PMC13710735; doi:10.1186/s41235-026-00747-5)
Supplement: Supplementary file 1 [file 41235_2026_747_MOESM2_ESM.pdf]

Study ID	Effect Size ID	N	Blocks / Prevalence	Manipulation	Design	Criterion Hedges' G	Sensitivity Hedges' G	Feedback	Stimuli	Observer type	Time Limit	Prevalence difference
Wolfe et al., 2007	exp 2	12	2 blocks: 200 trials (50%) then 1000 trials (2%)	A minimum RT was placed on target-absent responses	within-subjects	-4.216	-1.568	full	B	N	0	48
Russell & Kunar, 2012	exp 1 black/white	10	4 randomized blocks: 2 of 80 trials (50%) and 2 of 1000 trials (2%)	Blocks contained either colored or white stimuli. For colored blocks targets were always the same color	within-subjects	-2.330	0.305	none	L	N	1	48
Russell & Kunar, 2012	exp 1 colored	10	4 randomized blocks: 2 of 80 trials (50%) and 2 of 1000 trials (2%)	Blocks contained either colored or white stimuli. For colored blocks targets were always the same color	within-subjects	-2.987	0.760	none	L	N	1	48
Russell & Kunar, 2012	exp 2 majority	8	5 randomized blocks: 1 of 160 trials (50%) and 4 of 1000 trials (2%)	75% of targets were one color, 25% of targets were another color	within-subjects	-1.321	0.454	none	L	N	1	48
Russell & Kunar, 2012	exp 2 minority	8	5 randomized blocks: 1 of 160 trials (50%) and 4 of 1000 trials (2%)	75% of targets were one color, 25% of targets were another color	within-subjects	-1.682	0.700	none	L	N	1	48
Russell & Kunar, 2012	exp 3 tbox	9	6 blocks: 1 of 240 trials (50%), 5 of 1000 trials (2%)	Some trials contained a box: in Tbox trials, the target was within the box	within-subjects	-1.927	-0.449	none	L	N	1	48
Russell & Kunar, 2012	exp 3 dbox	9	6 blocks: 1 of 240 trials (50%), 5 of 1000 trials (2%)	Some trials contained a box: in Dbox trials, the target was outside the box	within-subjects	-2.093	0.592	none	L	N	1	48
Russell & Kunar, 2012	exp 3 no box	9	6 blocks: 1 of 240 trials (50%), 5 of 1000 trials (2%)	Some trials contained a box: in no box trials, a box was shown, but the target was absent	within-subjects	-2.146	0.634	none	L	N	1	48
Evans et al., 2011A	Boston	10	21 blocks: 20 of 100 trials (5%), 1 high prevalence (50%) at the halfway point	None	within-subjects	-2.931	-0.297	none	M	P	0	45
Evans et al., 2011B	Cardiff	12	21 blocks: 20 of 100 trials (2%), 1 high	None	within-subjects	-1.897	1.109	none	M	P	0	48

			prevalence (50%) at the halfway point									
Nakashima et al., 2013	radiologist bulla-gg	10	4 blocks: each of 500 trials (target 1: 40%, target 2: 8%, target 3: 2%)	Multiple targets of varying relative prevalence	within-subjects	-0.569	-1.890	none	M	P	2	32
Nakashima et al., 2013	novice bulla-gg	10	4 blocks: each of 500 trials (target 1: 40%, target 2: 8%, target 3: 2%)	Multiple targets of varying relative prevalence	within-subjects	-1.624	-2.272	none	M	N	2	32
Nakashima et al., 2013	radiologist bulla-canc er	10	4 blocks: each of 500 trials (target 1: 40%, target 2: 8%, target 3: 2%)	Multiple targets of varying relative prevalence	within-subjects	-0.199	-0.098	none	M	P	2	38
Nakashima et al., 2013	novice bulla-canc er	10	4 blocks: each of 500 trials (target 1: 40%, target 2: 8%, target 3: 2%)	Multiple targets of varying relative prevalence	within-subjects	-1.071	0.066	none	M	N	2	38
Nakashima et al., 2015	radiologist cancer	10	2 randomized blocks: 900 trials (2%) and 36 trials (50%)	None	within-subjects	-0.743	0.407	none	M	P	1	48
Nakashima et al., 2015	radiologist bulla	10	2 randomized blocks: 900 trials (2%) and 36 trials (50%)	None	within-subjects	-1.806	1.478	none	M	P	1	48
Nakashima et al., 2015	novice cancer	12	2 randomized blocks: 900 trials (2%) and 36 trials (50%)	None	WS	-2.052	1.925	none	M	N	1	48
Nakashima et al., 2015	novice bulla	12	2 randomized blocks: 900 trials (2%) and 36 trials (50%)	None	within-subjects	-2.181	1.790	none	M	N	1	48
Pinto & Papesh, 2024	exp 1	28	2 randomized blocks: 120 trials (50%) and 120 trials (25%)	None	within-subjects	-0.424	0.367	full	0	N	0	25
Pinto & Papesh, 2024	exp 2	34	2 randomized blocks: 90 trials (50%) and 90 trials (20%)	On some trials a gaze window limited view	within-subjects	-0.599	0.074	full	0	N	0	30
Goodhew	exp 1	380	2 randomized	Participants ranged in	within-	-1.031	0.006	none	0	N	0	46

& Edwards, 2024A			blocks: 400 trials (50%) and 400 trials (4%)	age from 20 to 80 years old	subjects							
Goodhew & Edwards, 2024B	exp 3	200	2 blocks: 400 trials (4%) and 400 trials (50%)	Visual search was part of task battery	within-subjects	-1.561	-0.0475	none	0	N	0	46
Thompson & Goodhew, 2021	exp 1-2	106 / 115	2 block: 400 trials (2%) and 400 trials (50%)	Visual search was part of task battery	between-subjects	-0.405	0.176	none	0	N	0	48
Van Wert et al., 2009	exp 1a	16	2 counterbalanced blocks: 200 trials (50%) and 1000 trials (2%)	None	within-subjects	-1.264	1.073	none	B	N	0	48
Van Wert et al., 2009	exp 2	16	2 counterbalanced blocks: 200 trials (50%) and 1000 trials (2%)	None	within-subjects	-1.464	0.250	none	B	N	0	48
Fleck & Mitroff, 2007	correctable	10	2 counterbalanced blocks: 200 trials (50%), and 1000 trials (2%)	None	within-subjects	-3.185	-0.609	none	O	N	0	48
Fleck & Mitroff, 2007	non-correctable	10	2 counterbalanced blocks: 200 trials (50%), and 1000 trials (2%)	None	within-subjects	-2.804	0.041	none	O	N	0	48
Buser et al., 2020	exp 1	71	2 counterbalanced blocks: 60 minutes (50%) and 60 minutes (12.5%)	Some participants were given a break during the task	within-subjects	-2.793	-0.841	full	B	P	1	37.5
Zsido et al., 2024	exp 1 snakes	28 / 30	3 blocks of 100 trials (10 or 50%)	None	between-subjects	-2.146	0.514	block	O	N	0	40
Zsido et al., 2024	exp 1 rabbits	28 / 29	3 blocks of 100 trials (10 or 50%)	None	between-subjects	-1.268	0.336	block	O	N	0	40
Zsido et al., 2024	exp 2 snakes	14 / 16	3 blocks of 100 trials (10 or 50%)	None	between-subjects	-1.812	0.111	block	O	N	0	40
Zsido et al., 2024	exp 2 rabbits	13 / 14	3 blocks of 100 trials (10 or 50%)	None	between-subjects	-1.410	0.637	block	O	N	0	40
Zsido et	exp 2	11 / 15	3 blocks of 100	None	between	-1.062	0.670	block	O	N	0	40

al., 2024	caterpillars		trials (10 or 50%)		-subjects							
Zsido et al., 2024	exp 2 cockroaches	13 / 13	3 blocks of 100 trials (10 or 50%)	None	between -subjects	-1.293	0.455	block	O	N	0	40
Schwark et al., 2012	exp 1 true feedback	10	2 counterbalanced blocks: 50 trials (50%) and 200 trials (4%)	None	within-subjects	-2.498	-1.443	full	L	N	1	46
Schwark et al., 2012	exp 2 true feedback	20	2 counterbalanced blocks: 100 trials (50%) and 300 trials (4%)	None	within-subjects	-2.024	-0.347	full	L	N	1	46
Kunar et al., 2017	exp 1 t/l	24	2 randomized blocks: 80 trials (50%) and 1000 trials (2%)	Mammogram and T/L blocks were intermixed	within-subjects	-1.299	1.039	none	L	N	1	48
Kunar et al., 2017	exp 1 mammograms	24	2 randomized blocks: 80 trials (50%) and 1000 trials (2%)	Mammogram and T/L blocks were intermixed	within-subjects	-1.201	1.292	none	M	N	1	48
Moher, 2019	exp 1-2	224	4 blocks: 2 of 100 trials (50%) and 2 of 100 trials (20%)	Some trials featured a salient distractor	between -subjects	-1.134	0.256	none	S	N	0	30
Kunar et al., 2021	exp 1a single	24	2 blocks: 1000 trials (2%) and 80 trials (50%)	None	within-subjects	-2.551	-0.024	none	M	N	1	48
Kunar et al., 2021	exp 1b single	24	2 blocks: 1000 trials (2%) 80 trials (50%)	None	within-subjects	-2.696	-0.112	none	M	N	1	48
Kang et al., 2023	exp 2 non-reward	34	6 blocks: 3 of 100 trials (20%) and 3 of 100 trials (50%)	None	within-subjects	-1.018	0.102	full	S	N	2	30
Kang et al., 2023	exp 2 reward	34	6 blocks: 3 of 100 trials (20%) and 3 of 100 trials (50%)	Participants were monetarily compensated for fast and correct responses	within-subjects	-1.298	0.371	full	S	N	2	30
Hebert et al., 2020	exp 1-2 single	16 / 16	18 blocks: 10 of 40 trials (50%) and 8 of 100 trials (10%)	5 display types were intermixed; only standard single display trials were included	between -subjects	-1.783	1.174	full	L	N	1	40
Miyazaki, 2015	monitored	100	2 blocks: 500 trials (2%), 100 trials	One condition was monitored with video	within-subjects	-4.046	0.264	full	O	N	1	48

			(50%) counterbalanced	cameras								
Miyazaki, 2015	not monitored	100	2 blocks: 500 trials (2%), 100 trials (50%) counterbalanced	One condition was monitored with video cameras	within-subjects	-4.165	0.279	full	O	N	1	48
Ishibashi & Kita, 2014	exp 1a	20	1 block of 300 trials: 96 "50% cue" trials and 204 "extreme cue (6%)" trials	Prevalence levels were intermixed in a single block	within-subjects	-2.290	-1.176	full	S	N	0	44
Ishibashi & Kita, 2014	exp 2	20	1 block of 600 trials: 192 "50% cue" trials and 408 "extreme cue (3%)" trials	Prevalence levels were intermixed in a single block	within-subjects	-3.131	-0.060	full	S	N	0	47
Corbett & Munneke, 2018	exp 1 mammograms	25	2 counterbalanced blocks: 400 trials (50%) and 400 trials (5%)	None	within-subjects	-1.478	-1.022	full	M	N	0	45
Corbett & Munneke, 2018	exp 2 tools	23	2 counterbalanced blocks: 360 trials (50%) and 360 trials (5%)	None	within-subjects	-2.668	-0.528	full	O	N	0	45
Madhavan et al., 2013	exp 1	36	4 blocks of 100 trials (50 or 20%)	None	between-subjects	-4.113	-0.299	full	B	N	2	30
Beanland et al., 2015	exp 1	18	2 blocks: 1500 trials (4%) and 120 trials (50%)	Objects varied in congruence and typicality with scenes	within-subjects	-0.830	2.091	none	C	N	1	46
Beanland et al., 2915	exp 2	20	2 blocks: 1500 trials (4%) and 120 trials (50%)	Objects varied in congruence and typicality with scenes	within-subjects	-1.126	1.363	none	C	N	1	46
Rich et al., 2008	set size 6	10	4 randomized blocks: 2 of 160 trials (50%) and 2 of 1000 trials (2%)	Set size varied within blocks	within-subjects	-2.807	0.339	full	L	N	2	48
Rich et al., 2008	set size 12	10	4 randomized blocks: 2 of 160 trials (50%) and 2 of 1000 trials (2%)	Set size varied within blocks	within-subjects	-2.898	0.182	full	L	N	2	48
Drew & Williams, 2017	exp 5-6	23 / 22	4 blocks: 2 of 60 (25%) and 2 of 60 (50%)	Showed observers least-examined locations in array	between-subjects	-0.257	0.575	none	C	N	0	25
Gillies &	exp 1	34	2 blocks: 50 trials	Similarity search	within-su	-2.103	-0.325	full	L	N	0	40

Kosovicheva, 2025			(50%) and 250 (10%) counterbalanced	followed by binary decision	bjects							
Gillies & Kosovicheva, 2025	exp 2	43	2 blocks: 50 trials (50%) and 300 (4%) counterbalanced	Similarity search followed by binary decision	within-subjects	-1.602	-0.034	full	L	N	0	46
Lyu et al., 2025	novice - feedback	26	1 block: 360 trials (first 120 50%, next 240 20%)	None	within-subjects	-2.667	0.218	full	M	N	0	30
Lyu et al., 2025	novice - no feedback	26	1 block: 360 trials (first 120 50%, next 240 20%)	None	within-subjects	0.113	0.096	none	M	N	0	30
Lyu et al., 2025	expert - feedback	24	1 block: 360 trials (first 120 50%, next 240 20%)	None	within-subjects	-3.366	0.266	full	M	P	0	30
Lyu et al., 2025	expert - no feedback	24	1 block: 360 trials (first 120 50%, next 240 20%)	None	within-subjects	0.190	0.039	none	M	P	0	30
