# Supplementary material for: The role of feedback in the low-prevalence effect: a meta-analysis of criterion shifts in visual search
Source: Cogn Res Princ Implic. 2026 Jul 29;11:55. doi: 10.1186/s41235-026-00747-5 (PMC13710735; doi:10.1186/s41235-026-00747-5)
Supplement: Supplementary file 2 [file 41235_2026_747_MOESM1_ESM.docx]

**Supplemental Materials**

**Table S1**: Results table for meta-regression models

| **Parameter** | **Moderator** | **Category** | **b** | **SE** | **95% CI Lower** | **95% CI Upper** | **t** | **dfs** | **p** | **m** | **k** |
| --- | --- | --- | --- | --- | --- | --- | --- | --- | --- | --- | --- |
| Criterion | Feedback | Block | -0.0862 | 0.232 | -0.595 | 0.423 | -0.372 | 11.3 | 0.717 | 40 | 17 |
| Criterion | Feedback | Full | -0.969 | 0.425 | -1.86 | -0.081 | -2.28 | 19.0 | 0.0341 | 56 | 28 |
| Criterion | Feedback | Full x low-prevalence level | -0.010 | 0.0531 | -0.136 | 0.116 | -0.189 | 6.84 | 0.855 | 56 | 28 |
| Criterion | Stimulus type | Simulated Baggage | -1.18 | 0.861 | -3.56 | 1.190 | -1.38 | 4.07 | 0.240 | 23 | 12 |
| Criterion | Stimulus type | Objects | -0.209 | 0.471 | -1.26 | 0.843 | -0.444 | 9.81 | 0.667 | 36 | 16 |
| Criterion | Stimulus type | Shapes | -0.222 | 0.552 | -1.99 | 1.543 | -0.402 | 2.97 | 0.715 | 23 | 11 |
| Criterion | Stimulus type | Letters | -0.543 | 0.258 | -1.15 | 0.0682 | -2.11 | 6.88 | 0.0737 | 33 | 13 |
| Criterion | Observer expertise | Professionals | 0.186 | 0.294 | -0.554 | 0.926 | 0.632 | 5.39 | 0.553 | 62 | 29 |
| Criterion | Observer expertise | Professionals (medical only) | 0.054 | 0.356 | -0.90 | 1.008 | 0.151 | 4.42 | 0.886 | 18 | 8 |
| Criterion | Time limit | 8-12 seconds | -0.659 | 0.369 | -1.47 | 0.154 | -1.79 | 10.92 | 0.102 | 49 | 24 |
| Criterion | Time limit | <6 seconds | -0.139 | 0.436 | -1.21 | 0.929 | -0.319 | 5.99 | 0.760 | 43 | 21 |
| Criterion | Prevalence difference | Continuous | -0.0543 | 0.0185 | -0.096 | -0.0132 | -2.94 | 10.04 | 0.0147 | 62 | 29 |
| Sensitivity | Feedback | Block | 0.140 | 0.179 | -0.267 | 0.546 | 0.784 | 8.76 | 0.454 | 40 | 17 |
| Sensitivity | Feedback | Full | -0.463 | 0.243 | -0.972 | 0.0453 | -1.91 | 18.7 | 0.0718 | 56 | 28 |
| Sensitivity | Feedback | Full x low-prevalence level | 0.0370 | 0.0210 | -0.0132 | 0.0872 | 1.77 | 6.59 | 0.123 | 56 | 28 |
| Sensitivity | Stimulus type | Simulated Baggage | -0.507 | 0.644 | -2.238 | 1.22 | -0.788 | 4.37 | 0.471 | 23 | 12 |
| Sensitivity | Stimulus type | Objects | 0.0815 | 0.308 | -0.611 | 0.774 | 0.265 | 9.34 | 0.797 | 36 | 16 |
| Sensitivity | Stimulus type | Shapes | -0.216 | 0.427 | -1.561 | 1.13 | -0.505 | 3.06 | 0.648 | 23 | 11 |
| Sensitivity | Stimulus type | Letters | 0.0546 | 0.437 | -0.979 | 1.09 | 0.125 | 6.99 | 0.904 | 33 | 13 |
| Sensitivity | Observer expertise | Professionals | -0.333 | 0.407 | -1.431 | 0.766 | -0.817 | 4.32 | 0.457 | 62 | 29 |
| Sensitivity | Observer expertise | Professionals (medical only) | -0.429 | 0.490 | -1.76 | 0.90 | -0.875 | 4.26 | 0.428 | 18 | 8 |
| Sensitivity | Time limit | 8-12 seconds | 0.252 | 0.261 | -0.324 | 0.827 | 0.965 | 10.84 | 0.356 | 49 | 24 |
| Sensitivity | Time limit | <6 seconds | 0.0988 | 0.411 | -0.914 | 1.112 | 0.240 | 5.84 | 0.818 | 43 | 21 |
| Sensitivity | Prevalence difference | Continuous | 0.0107 | 0.0119 | -0.0159 | 0.0372 | 0.893 | 10.17 | 0.393 | 62 | 29 |
